# MR Imaging of Uterine Epithelioid Trophoblastic Tumor: A Case Report

**DOI:** 10.2463/mrms.cr.2015-0070

**Published:** 2016-03-21

**Authors:** Sakiko KAGEYAMA, Masafumi KANOTO, Yukio SUGAI, Takeshi SUTO, Satoru NAGASE, Mitsumasa OSAKABE, Takaaki HOSOYA

**Affiliations:** 1 Department of Diagnostic Radiology, Yamagata University School of Medicine, 2-2-2 Iida-Nishi, Yamagata 990-9585, Japan; 2 Department of Gynecology, Yamagata University School of Medicine; 3 Department of Diagnostic Pathology, Yamagata Prefectural Central Hospital

**Keywords:** epithelioid trophoblastic tumor, MRI, uterus, necrosis, hemorrhage

## Abstract

Epithelioid trophoblastic tumor (ETT) is a rare gestational trophoblastic neoplasm of chorionic-type intermediate trophoblasts, and it is most frequently located in the lower uterine segment and endocervix. Due to the epithelial-growth pattern with geographic necrosis exhibited by the neoplastic cells, ETT is commonly confused, both clinically and pathologically, with squamous cell carcinoma. Although there have been no previous reports of ETT focusing on computed tomography (CT) or magnetic resonance imaging (MRI) findings, we report a case of uterine ETT with special attention to the MRI findings referring to the pathological findings and MR images of previous reports. A 42-year-old Japanese woman (gravid 1, para 1) presented with uterus enlargement during screening, and complained of recent-onset lower abdominal pain. The MRI showed a solid tumor throughout the entire myometrium of the lower uterine segment, with the hemorrhagic cystic portion extending to the posterior subserosal space. Following hysterectomy, the final pathological diagnosis was ETT. An ETT is essentially a solid tumor composed of intermediate trophoblasts that exhibit an epithelial-like growth pattern and contain geographic necrosis with calcification. In our case, MRI revealed a non-specific-intensity solid tumor in the lower uterine segment with massive necrosis and hemorrhage extending to the subserosa. While it is difficult to distinguish between ETT and uterine carcinomas, recognition of certain tumor shapes and necrosis could enable more accurate diagnosis before treatment.

## Introduction

Epithelioid trophoblastic tumor (ETT) is a rare gestational trophoblastic neoplasm of chorionic-type intermediate trophoblasts.^1^ In 1998, Shih and Kurman established ETT as a distinct entity within the category of gestational trophoblastic disease.^2^ ETT is distinguished from, but may occur in conjunction with, placental site trophoblastic tumor and choriocarcinoma.^3^ The most frequent location of ETT is the lower uterine segment and endocervix. As the neoplastic cells in ETT exhibit an epithelial-like growth pattern with geographic necrosis, ETT is commonly confused, both clinically and pathologically, with squamous cell carcinoma.^1,2,4^

An ETT is generally a slow-growing malignant tumor, though some cases have had aggressive and fatal clinical courses involving multiple metastases. Over 100 cases have been reported so far, many of them in clinical or pathological reports. No previous report has focused on the CT or MRI findings associated with ETT. Referring to pathological findings and MR images of previous reports, we report a case of uterine ETT with special attention to the MRI findings.

## Case Presentation

A 42-year-old Japanese woman, gravid 1, para 1 (normal vaginal delivery 13 years previously), presented uterine enlargement in screening. She complained of recent-onset lower abdominal pain and a few months’ history of urinary incontinence and constipation.

In a pelvic examination, the uterus and left ovary presented as a fist-size lump. There were no abnormal findings in the vulva or vagina. Because the portio could not be clearly identified, endometrial smear test and uteroscopy were not possible. Serum tumor markers including carcinoembryonic antigen (CEA), CA125, CA19-9, and squamous cell carcinoma (SCC) were all within the normal range. Human chorionic gonadotropin (hCG) was not measured prior to operation.

**Fig. 1.**
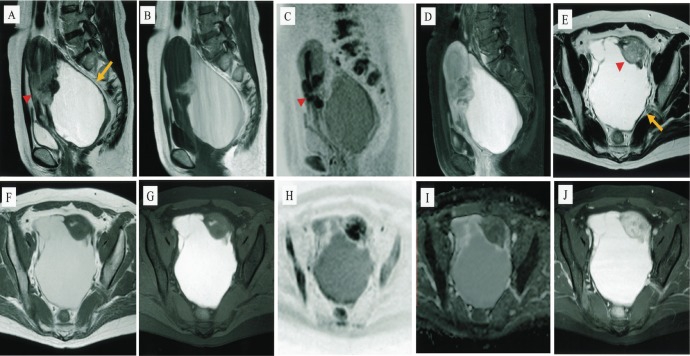
Magnetic resonance imaging findings. (**A**, **E**): T_2_-weighted images, (**B**, **F**) T_1_-weighted images, (**G**) fat suppression T_1_-weighted images, (**C**, **H**) diffusion-weighted images (DWIs), (**I**) apparent diffusion coefficient value map, (**D**, **J**) gadolinium-DTPA enhanced T_1_-weighted images. Solid mass in uterus (arrowhead) was hyperintense on T_2_WI, isointense on T_1_WI, heterogeneously enhanced on gadolinium-DTPA-enhanced T_1_WI, and hyperintense on DWI. Its apparent diffusion coefficient value was 0.9 × 10^−3^ mm^2^/s. The cystic portion (arrow) had an irregular wall and a septum protruding into the solid mass of the posterior wall. Its contents appeared to be old hemorrhagic matter with precipitated clot-like material. DTPA, diethylene triamine pentaacetic acid.

Ultrasound revealed a cystic mass 125 × 75 mm in diameter behind the uterus; this was interpreted as an endometrioid cyst. The MRI showed a solid lesion extending throughout the myometrium of the lower uterine segment and an irregular large cystic lesion filling the pelvic cavity behind the uterus (Fig. 1). The solid lesion in the uterus was hyperintense on T_2_-weighted images (T_2_WIs) [repetition time (TR)/echo time (TE) = 1300/90 ms], isointense on T_1_-weighted images (T_1_WIs) (TR/TE = 500/12) and heterogeneously enhanced on gadolinium-diethylene triamine pentaacetic acid (DTPA)-enhanced T_1_WI. It appeared hyperintense in diffusion-weighted images (DWIs) (b = 1000), with an apparent diffusion coefficient (ADC) value of 0.9 × 10^−3^ mm^2^/s. The subserosal cystic lesion had an irregular wall and septum, and protruded into the solid lesion of the posterior wall like an ulceration. The contents of the cystic lesion appeared hyperintense on T_2_WI, T_1_WI, and fat suppression T_1_WI, and included T_2_WI-hypointense precipitated material. Slightly solid components were present on the inner surface of the cystic wall. There were no findings indicating adenomyosis in the myometrium of the uterus, and no abnormal findings in the endometrium and left ovary. The right ovary was not detected. To summarize, the tumor was a solid mass of non-specific intensity replacing the myometrium of the lower uterine segment with a large subserosal hemorrhagic cystic lesion; unfortunately, however, the solid mass had not been observed initially. The operation was performed as for an endometriotic cyst of the ovary or peritoneum.

**Fig. 2.**
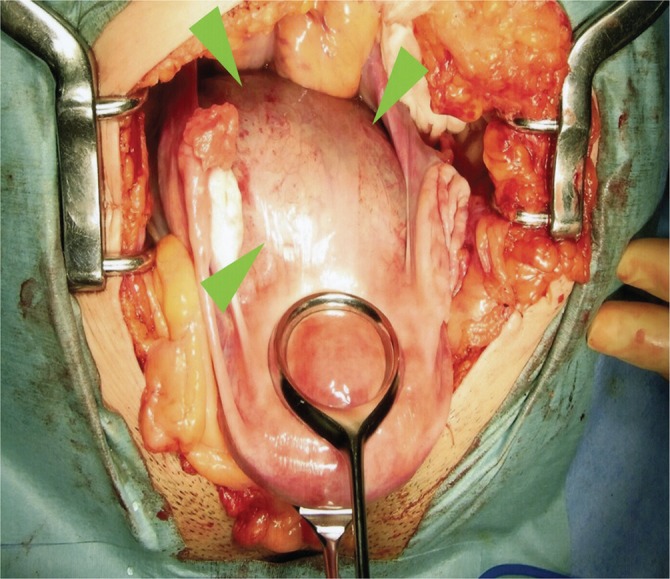
Surgical findings. The cystic portion was observed under the border ligament (arrowheads).

Intraoperative observation revealed a subserosal large mass (Fig. 2), and a hysterectomy was performed. It was a cystic mass containing old hemorrhagic matter, with a fragile, yellowish, solid mass that extended deep within the cystic mass. Exploring further, the surgeons reached the uterine cavity in the lower uterine segment. There were no abnormal findings in the ovary. The solid mass was diagnosed mid-operation as poorly differentiated carcinoma. Accordingly, bilateral salpingo-oophorectomy and lymphadenectomy were performed in addition to hysterectomy.

**Fig. 3.**
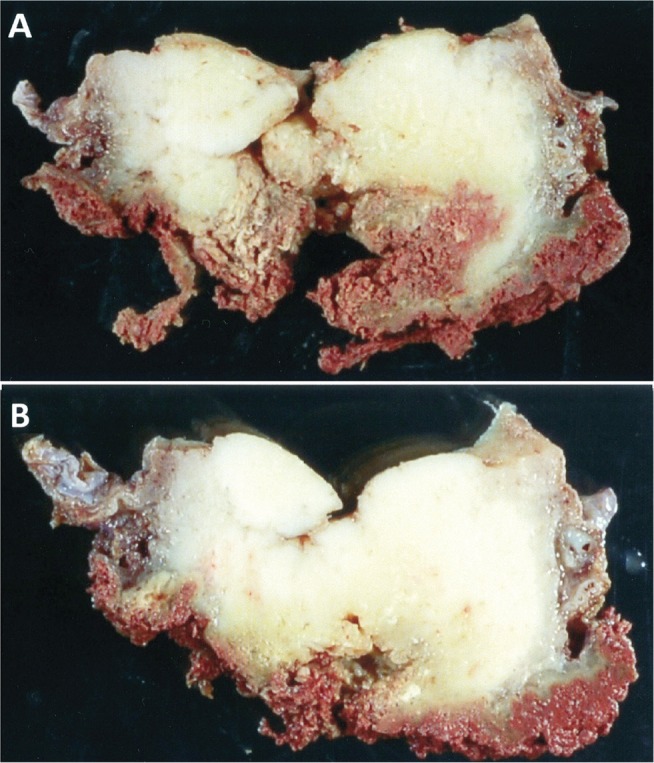
Resected specimens of posterior wall (macroscopic, short axis). (**A**) uterine cervix and (**B**) lower uterine segment. A solid yellow mass involved the entire thickness of the myometrium; beneath it was a quantity of brown hemorrhagic and necrotic tissue within a cystic lesion. The surface of the uterine cavity was smooth. The cystic lesion was opened to the uterine cavity of the cervix through the posterior solid mass.

**Fig. 4.**
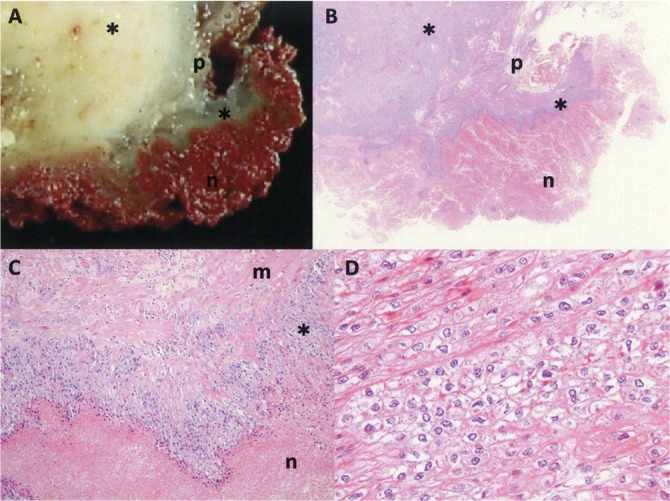
Pathological findings. (**A**) Gross (×0.39), (**B**) HE (×0.39), (**C**) HE (×10), (**D**) tumor HE (×40). A solid yellow mass (*) involved the entire thickness of the myometrium. Necrotic tissue was present under the thinly stretched myometrium, and layers of tumor were observed between the myometrium and the necrotic tissue. There was no cystic wall. The mass was composed of nests and cords of intermediate trophoblasts and contained characteristic geographic necrosis. HE, hematoxylin and eosin; m, myometrium; n, necrotic and hemorrhagic tissue; p, parametrium.

Pathologically, the carcinoma specimen was a solid yellow mass involving the entire thickness of the myometrium in the lower uterine segment (Fig. 3). There were no abnormal findings in the endometrium. The cystic lesion was opened to the uterine cavity through the posterior solid mass, as MRI had shown, but it is uncertain whether this was due to perforation during the operation or to fistula formation. The uterine solid mass was composed of intermediate trophoblasts showing an epithelial-like growth pattern; it also contained geographic necrosis with calcification. The cystic lesion extending into the subserosa contained massive amounts of necrotic tissue and old hemorrhagic matter. Necrotic tissue was observed under the thinly stretched myometrium, and layers of tumor were present between the myometrium and the necrotic tissue (Fig. 4), where no cystic wall was present. No endometriotic tissue was found in either the myometrium or the tumor, though massive necrosis and hemorrhage was observed. The final diagnosis was ETT.

A follow-up CT 3 months after the operation revealed multiple lung metastases. Chemotherapy and CyberKnife were administered to treat these. Today, more than 4 years after the operation, the patient is alive and still undergoing treatment.

## Discussion

ETT is a rare disease, so few studies have reported on the radiographic findings seen in uterine ETT. Although there is no previous report focused on CT or MRI findings, and only PET-CT findings has been reported,^5^ there are three clinical or pathological reports that contained MR images.^5–7^ This report aimed to identify some keys to the presurgical differential diagnosis of ETT, namely, CT or MRI findings that would be incompatible with a diagnosis of uterine carcinoma, referring to pathological findings and MR images of previous reports.

The present case consisted of a solid tumor in the lower uterine segment containing massive necrosis and hemorrhagic matter and extending to the subserosa. The solid tumor had nonspecific signal intensity on MRI.

In three cases containing MR images, T_2_WI was available for two cases and enhanced image was available for one case. A case reported by Noh et al. showed T_2_WI-hyperintense solid tumor with cystic hemorrhage into subserosa.^6^ It was quite similar findings to those in the present case. Kara et al. have reported a case of uterine ETT diagnosed by fractional curettage, it was difficult to detect tumor as its intensity was similar to that of a normal endometrium.^5^ Last case was well-circumscribed tumor containing hypoenhanced area reported by Okumura et al., and it was diagnosed ETT with necrotic cell debris.^7^

In three cases, including the present case, the signal intensity of ETT shows hyperintense on T_2_WI as a non-specific malignant tumor.^5,6^ Generally, in its outward appearance, ETT is essentially a white solid mass. Histologically, it is composed of nests and cords of intermediate trophoblasts, shows an epithelial-like growth pattern, and contains characteristic geographic necrosis with calcification.^2^ This MRI finding is consistent that ETT does not contain specific-intensity component such as fibrous tissue and abnormal finding. Accordingly, it was difficult in this case, as in similar cases, to distinguish between ETT and uterine carcinoma based on signal intensity data only.

In addition, the present case had massive necrosis and hemorrhage. Since the first report on ETT by Shih and Kurman, many cases have been reported to contain various amounts of necrosis and hemorrhage, which appear to be independent of tumor size. In fact, necrosis and hemorrhage seem to have fairly common characteristics of ETT.^2,6–14^ Shen et al. have reported nine cases of ETT, seven of which had extensive massive necrosis and two of which had focal necrosis in the absence of chemotherapy.^8^ Lo et al. reported a 9-cm ETT extending to the right pelvic side wall, with an extensive area containing necrotic matter.^9^ Liang et al., Noh et al., and Fadare et al. have reported four cases of large cystic lesions containing hemorrhagic material and necrosis, as in this case, and some of their cases had fistulas in the uterine cavity.^6,10,11^ In the present case, the fistula was narrow and was associated with no inflammation or foreign body reaction along its length; an inflow of menstrual blood through it was therefore considered unlikely.

Retrospectively, the differential diagnosis of uterine ETT is similar to that of subserosal cystic lesions as well as endometriotic cyst on an ovary or the peritoneum and cystic adenomyosis. The existence of uterine tumors and the absence of adenomyosis in the myometrium, however, are not typically associated with these other diagnoses.

Although it does contain some calcifications in the geographic necrosis, these are apparently too small and too low in density to be recognized on CT. In our case, pelvic CT was not performed before the operation, but we were unable to find any calcifications in the metastatic lung tumor on follow-up CT.

Turning to the shape and macroscopic growth pattern of uterine ETT, it usually presents as a circumscribed mass of various size, the maximum tumor diameter ranged from 0.5 to 14.8 cm,^1,15–17^ but sometimes presents as an invasive growth. Several shapes of ETT have been reported, including solitary nodules of the myometrium,^4,7,11,12^ a solid mass replacing the myometrium,^6^ polypoid lesions extending into the uterine cavity,^4,11^ and irregular lesions along cesarean section scars.^3^ Accordingly, it might be difficult to distinguish ETT from uterine carcinomas and intramural uterine myomas based on shape alone. Especially in the case of a tumor extending into the uterine cavity, a differential diagnosis cannot be successfully made. A circumscribed intramural uterine lesion with malignant tumor intensity on MRI, on the other hand, is unlikely to be a common uterine carcinoma, excepting those based upon ectopic endometrium. Though it is not a specific finding, the frequent appearance of necrosis and hemorrhage in conjunction with ETT merit their consideration in the differential diagnosis of uterine carcinomas.

ETT is a rare disease that is not often seen in clinical practice. There is as yet no standard treatment for it; rather, it is treated as a gestational trophoblastic neoplasm. The ability to diagnose ETT accurately prior to treatment would be useful. Though ETT’s non-specific intensity on MRI contributes nothing to the diagnostic process, gross necrosis and hemorrhage are not uncommon, to the best of our knowledge. Therefore, whereas it may be difficult to diagnosis ETT, if there is an intramural lower uterine mass with massive necrosis and hemorrhage, ETT is considered to one of the differential diagnoses of uterine carcinoma. This is only one case report referencing previous clinical reports, and further reports in more patients are needed.
